# Nemolizumab as an Alternative Therapeutic Option for Indolent Systemic Mastocytosis

**DOI:** 10.7759/cureus.101086

**Published:** 2026-01-08

**Authors:** Sezim Minbaeva, Stephen K Tyring

**Affiliations:** 1 Department of Dermatology, Texas Tech University Health Sciences Center School of Medicine, Lubbock, USA; 2 Department of Dermatology, The University of Texas Health Science Center at Houston, Houston, USA

**Keywords:** anti-il-31 therapy, indolent systemic mastocytosis, mast cell disease, nemolizumab, systemic mastocytosis, systemic mastocytosis therapy

## Abstract

Indolent systemic mastocytosis (ISM) is a clonal mast cell disorder characterized by recurrent symptoms that can persist despite treatment with antihistamines, leukotriene antagonists, and mast cell stabilizers. Treatment options are limited when symptoms remain refractory, and avapritinib is contraindicated or declined. We describe the case of a 62-year-old female with an 11-year history of ISM who developed worsening pruritus and urticaria despite extensive conservative and biologic therapies, including topical corticosteroids, hydroxyzine, montelukast, omalizumab, and dupilumab. Given her history of stroke, she declined avapritinib. Nemolizumab was initiated with a 60 mg loading dose followed by 30 mg every four weeks. After 12 doses, she achieved complete resolution of pruritus. This report suggests that nemolizumab may represent a potential therapeutic option for refractory ISM when standard therapies fail or targeted KIT inhibition is not feasible.

## Introduction

Mastocytosis comprises a heterogeneous group of neoplastic disorders characterized by the abnormal proliferation and accumulation of mast cells (MCs) in various tissues [1,2]. Somatic mutations in the KIT gene, especially the KIT D816V variant, have been implicated in the constitutive activation of the KIT receptor tyrosine kinase, which drives MC proliferation [1-3]. The clinical presentation of mastocytosis ranges from skin-limited forms (cutaneous mastocytosis) to extracutaneous disease with multi-organ involvement (systemic mastocytosis) [1,2].

Indolent systemic mastocytosis (ISM) is the most common subtype in adulthood, comprising approximately 80-90% of all systemic mastocytosis diagnoses [3]. Common clinical presentations of ISM include flushing, pruritus, urticaria, gastrointestinal symptoms, and recurrent anaphylaxis [1,2,4]. ISM is diagnosed based on the WHO criteria for systemic mastocytosis, which require either one major criterion of multifocal dense mast cell infiltrates in the bone marrow or another extracutaneous organ plus one minor criterion, or at least three minor criteria alone. Minor criteria include more than 25% atypical or spindle-shaped mast cells, the presence of a KIT D816V mutation, an aberrant mast cell immunophenotype (CD25, CD2, CD30), and persistently elevated serum tryptase of ≥20 ng/mL. ISM is distinguished from advanced systemic forms by the absence of C findings, which indicate organ damage caused by mast cell infiltration.

ISM is generally managed with symptom-directed therapy, including H1 and H2 antihistamines, leukotriene receptor antagonists, and mast cell stabilizers. For moderate-to-severe cases refractory to mediator-targeted therapy, KIT inhibition has emerged as an important therapeutic option. Low-dose avapritinib, which targets KIT D816V, is now FDA-approved for symptomatic ISM and has been shown to produce significant reductions in disease burden [1,5]. Contraindications for avapritinib include severe thrombocytopenia, with platelet counts below 50 × 10⁹/L, due to an increased risk of bleeding [2]. We report the first case of ISM treated with nemolizumab, which resulted in complete resolution of pruritus. A comprehensive literature search of PubMed, Scopus, and Embase found no prior reports of nemolizumab use in this condition.

## Case presentation

A 62-year-old Caucasian female presented with a case of ISM in February 2025. She had a past medical history of multiple mastocytomas confirmed via skin biopsy. Over the past 11 years, she had continued to develop symptoms of weakness, flushing, pruritus, chronic urticaria, angioedema, edematous plaques, nausea, loose stools, constipation, and musculoskeletal pain, with confirmed bone lesions on a 2015 CT scan performed by an outside physician. She had slightly elevated tryptase levels in April 2014 (11 ng/mL) and in February 2016 (13 ng/mL). Despite conservative treatment with topical corticosteroids, montelukast, and hydroxyzine, the patient continued to experience symptoms, including progression and worsening of pruritus and urticaria. A series of omalizumab 300 mg injections was subsequently started in 2014 at four-week intervals but was discontinued after eight injections due to insurance limitations. At that time, mild improvement was noted with combination therapy consisting of topical corticosteroids, montelukast, hydroxyzine, and omalizumab injections.

In November 2022, the patient presented with worsening mastocytosis and urticaria involving the face, neck, trunk, and bilateral arms and legs. She had discontinued prior treatment after not being seen in the office for the previous two years. At that time, given her past medical history, she was restarted on topical corticosteroids, hydroxyzine, and dupilumab 300 mg/2 mL injections at two-week intervals. Dupilumab was used instead of omalizumab due to insurance coverage and demonstrated continued improvement over the course of three months with six injection cycles. Dupilumab was ultimately discontinued in December 2022 after she declined further treatment due to a history of stroke, but she continued to use hydroxyzine and topical corticosteroids as needed. The patient trialed doxepin in November 2023 without success.

**Figure 1:**
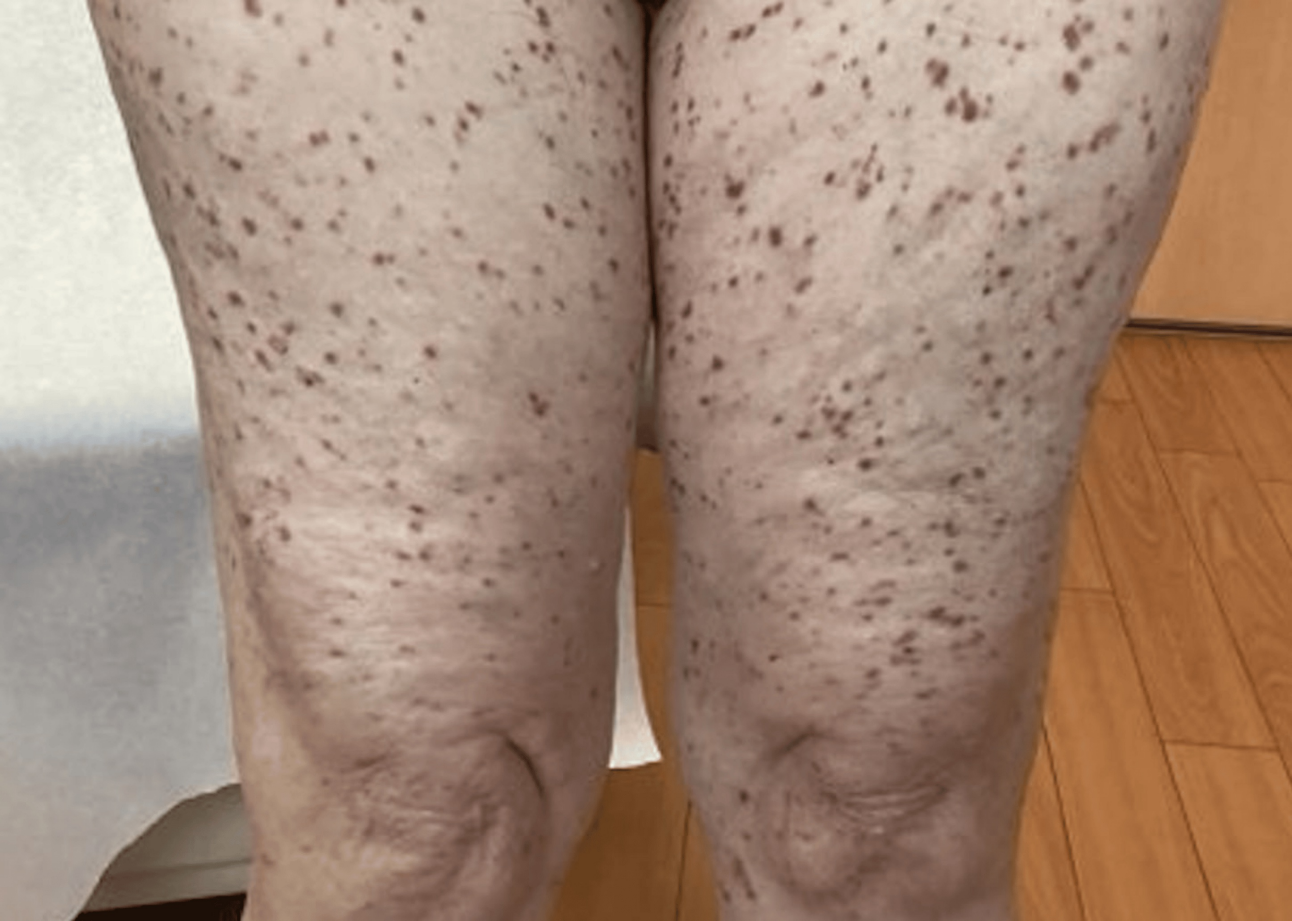
Baseline clinical presentation showing mastocytosis involving bilateral legs

**Figure 2:**
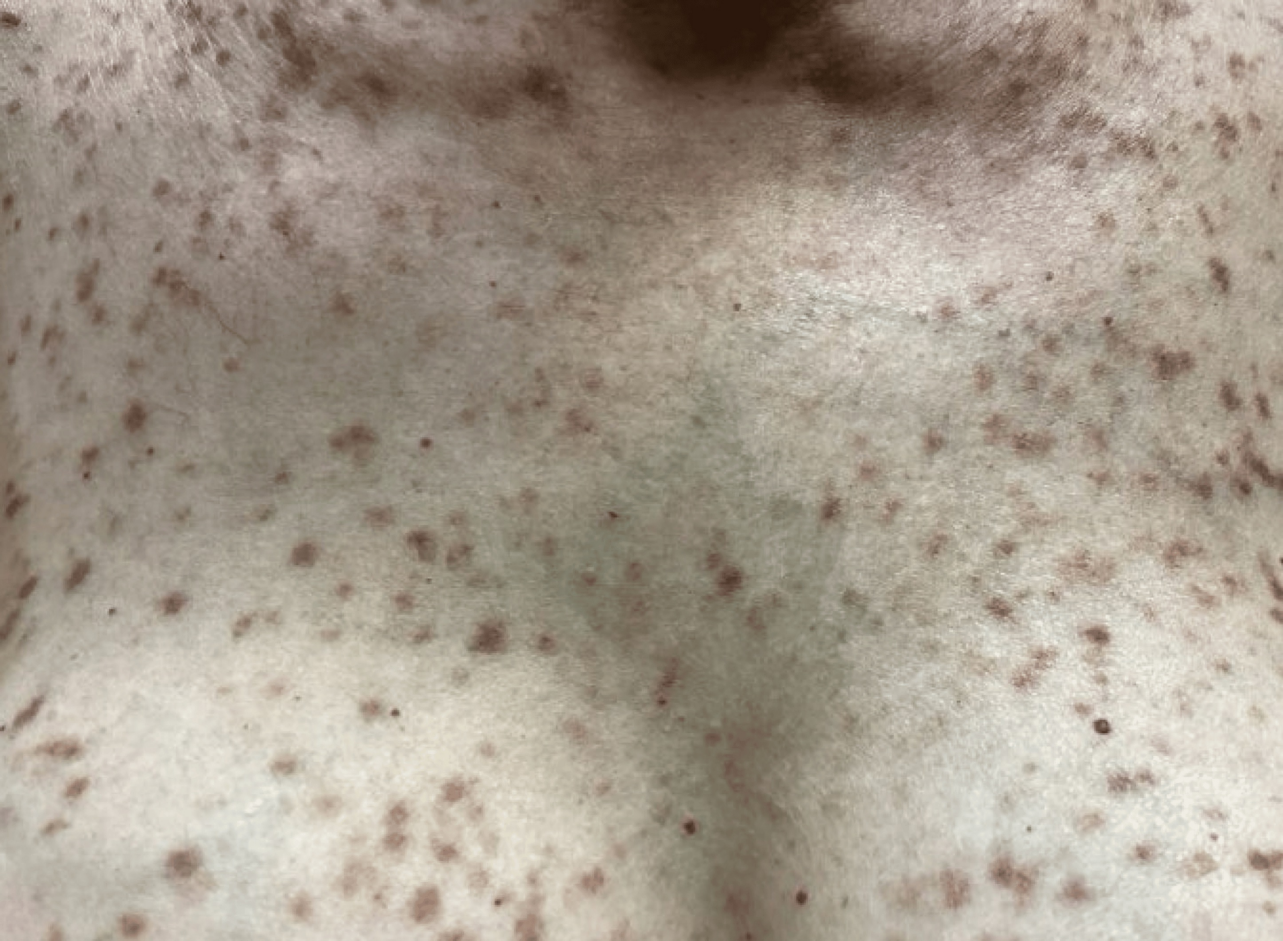
Baseline clinical presentation showing mastocytosis involving the chest

**Figure 3:**
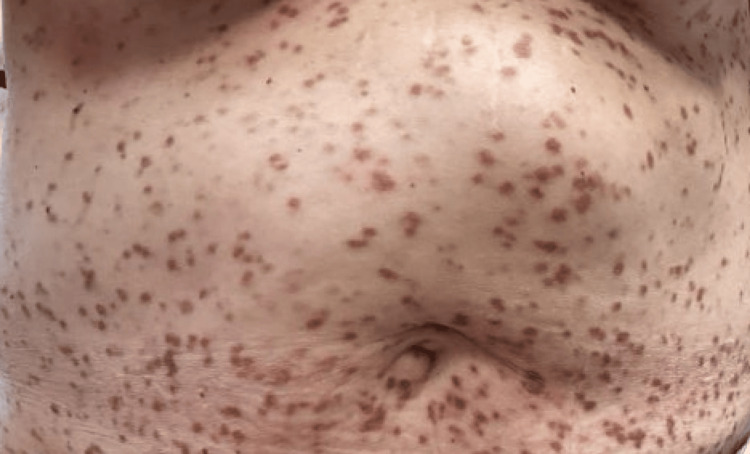
Baseline clinical presentation showing mastocytosis involving the abdomen

**Figure 4:**
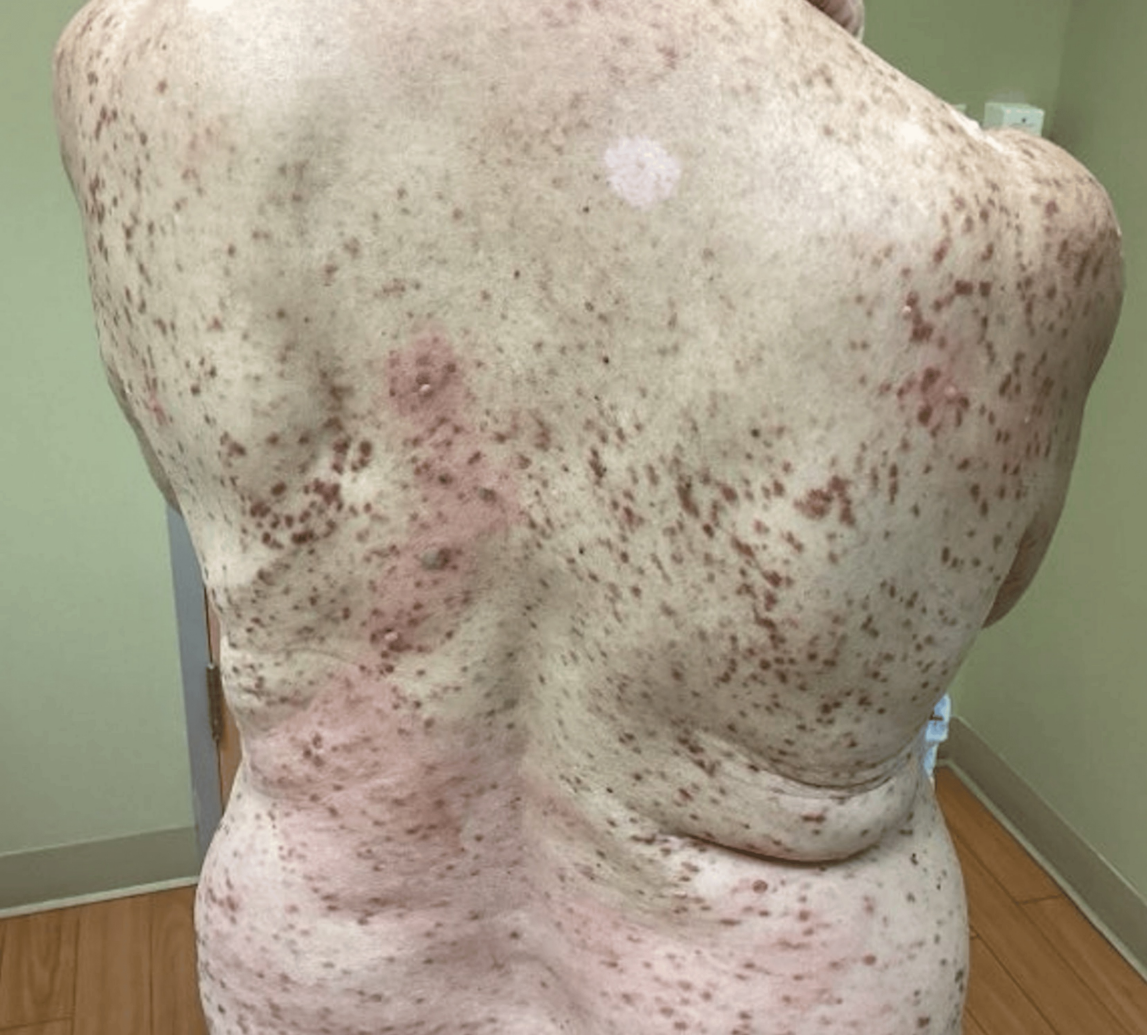
Baseline clinical presentation showing mastocytosis involving the upper and lower back with a positive Darier sign

In February 2025, the patient again presented with worsening mastocytosis (>20 nodules) (Figures 1-3) with a positive Darier sign along the chest and back (Figure 4). She reported that she was unable to function socially or professionally and was physically impaired due to her condition. At this time, because she had failed to respond to previous topical corticosteroids and systemic therapy, nemolizumab was initiated with an initial subcutaneous dose of 60 mg administered on day zero, followed by 30 mg subcutaneously every four weeks. She continued oral hydroxyzine and topical corticosteroids as needed and continued to decline avapritinib treatment due to her stroke history.

The patient continued to receive nemolizumab injections at four-week intervals between February 2025 and November 2025 (12 total injections) and was monitored for injection-site reactions at each visit. Of note, the patient had elevated tryptase levels of 39.1 ng/mL in April 2025, indicating an increased systemic mast cell burden compared with the last tryptase measurement in 2016, but did not have any notable mutations in the PDGFRa gene exons 5, 6, 11, 12, 14, 16, and 18 (Table 1). Upon presentation in November 2025, the patient had complete resolution of pruritus after nemolizumab treatment, which had not been achieved within the past 11 years. At this time, she denied any issues with free bleeding, healing, or scarring but continued to have resolving erythematous nodules over the chest, legs, and arms. A negative Darier sign was documented, and the patient denied urticaria when her clothing contacted her mastocytomas.

**Table 1:** Longitudinal serum tryptase levels in 2014, 2016, and 2025

Month/year of collection	Serum tryptase (ng/mL)	Reference range (ng/mL)
4/2014	11	2–10
2/2016	13	<11
4/2025	39.1	<11

## Discussion

ISM is a rare disease, affecting approximately 10 adults per 100,000 individuals [3]. Although overall mortality is close to normal, it has been associated with a significant symptom burden due to abnormal mast cell proliferation [1,2]. The impact of ISM is not limited to acute degranulation events but also involves a persistent, multisystem effect. Pruritic symptoms and chronic urticaria may disrupt daily function, making it difficult for patients to focus and complete everyday tasks. Gastrointestinal symptoms may further impair social functioning and interfere with meeting nutritional goals, while musculoskeletal pain may limit overall mobility, particularly in the elderly population [1,2,4]. Avapritinib is the current FDA-approved treatment for ISM that is refractory to conservative and symptom-focused therapies. However, this medication may be contraindicated in patients with thrombocytopenia and a heightened risk of free bleeding [2]. In cases like this patient, avapritinib use may be refused due to the patient’s medical history.

Nemolizumab is a human IgG2 monoclonal antibody that selectively targets the interleukin-31 receptor A (IL-31RA), thereby inhibiting IL-31 signaling. IL-31 has been implicated in chronic pruritus and skin inflammation, and by blocking this pathway, nemolizumab provides a mechanistically sound approach to symptom control in patients with ISM [6,7]. Clinical studies have demonstrated its efficacy in adults with prurigo nodularis and in adults and children aged 12 years and older with atopic dermatitis [6,7]. Greater reductions in pruritus have been noted when using nemolizumab in conjunction with topical agents for atopic dermatitis [8]. Nemolizumab is generally well-tolerated, but common adverse effects include injection site reactions, peripheral edema, nausea, and diarrhea, whereas rare but serious effects may include hypersensitivity reactions, asthma exacerbations, and infections [8].

Our patient experienced complete pruritus resolution following nemolizumab therapy, administered as a 60 mg subcutaneous dose on day zero, followed by 30 mg every four weeks for a total of 12 injections. Although a bone marrow biopsy was not performed, the patient’s clinical features, including persistent cutaneous mastocytosis, mediator-related symptoms (pruritus, flushing, urticaria, gastrointestinal symptoms), and elevated serum tryptase (39 ng/mL), are consistent with WHO minor criteria for systemic mastocytosis. The absence of C findings, including cytopenias, organ dysfunction, malabsorption, and pathologic fractures, supports classification as ISM. Throughout the treatment period, she continued concomitant use of antihistamines and topical corticosteroids, which may have contributed as a confounding factor when interpreting the observed treatment response. She reported no adverse effects, and the therapy was well tolerated. This report highlights nemolizumab as a potential alternative treatment for refractory ISM in patients who are unable or unwilling to undergo avapritinib therapy.

## Conclusions

ISM is a rare, clonal mast cell disorder that typically occurs in the adult population. ISM can be challenging to treat, especially in cases that do not respond to standard therapies, including antihistamines, leukotriene receptor antagonists, and mast cell stabilizers. This report highlights the potential of nemolizumab as an effective treatment option for refractory ISM. Further research is needed to establish the optimal dosage, ideal patient populations, and the long-term efficacy and safety of nemolizumab for ISM.

## References

[1] Pardanani A (2023). Systemic mastocytosis in adults: 2023 update on diagnosis, risk stratification and management. Am J Hematol.

[2] Syal A, Toh J, McInerney A, Tremblay D (2025). The evaluation, management, and future of indolent systemic mastocytosis. Ann Hematol.

[3] Tiryaki TO, Özkan SG, Erdem S (2023). Comprehensive mastocytosis data analysis from a single center. BMC Cancer.

[4] Sizemore JA, Hansen S, Moran CA, Petty AJ, Rein LA, Jamison MO, Nicholas MW (2025). Mastocytosis: part i: pathogenesis, clinical presentation and classification. J Am Acad Dermatol.

[5] Gotlib J, Gerds AT, Abdelmessieh P (2024). NCCN Guidelines® Insights: Systemic Mastocytosis, Version 3.2024. J Natl Compr Canc Netw.

[6] Kabashima K, Matsumura T, Komazaki H, Kawashima M (2020). Trial of nemolizumab and topical agents for atopic dermatitis with pruritus. N Engl J Med.

[7] Kwatra SG, Yosipovitch G, Legat FJ (2023). Phase 3 trial of nemolizumab in patients with prurigo nodularis. N Engl J Med.

[8] Silverberg JI, Wollenberg A, Reich A (2024). Nemolizumab with concomitant topical therapy in adolescents and adults with moderate-to-severe atopic dermatitis (ARCADIA 1 and ARCADIA 2): results from two replicate, double-blind, randomised controlled phase 3 trials. Lancet.

